# Simple Measurement of IgA Predicts Immunity and Mortality in Ataxia-Telangiectasia

**DOI:** 10.1007/s10875-021-01090-8

**Published:** 2021-09-03

**Authors:** Stefan Zielen, Ruth Pia Duecker, Sandra Woelke, Helena Donath, Sharhzad Bakhtiar, Aileen Buecker, Hermann Kreyenberg, Sabine Huenecke, Peter Bader, Nizar Mahlaoui, Stephan Ehl, Sabine M. El-Helou, Barbara Pietrucha, Alessandro Plebani, Michiel van der Flier, Koen van Aerde, Sara S. Kilic, Shereen M. Reda, Larysa Kostyuchenko, Elizabeth McDermott, Nermeen Galal, Claudio Pignata, Juan Luis Santos Pérez, Hans-Juergen Laws, Tim Niehues, Necil Kutukculer, Markus G. Seidel, Laura Marques, Peter Ciznar, John David M. Edgar, Pere Soler-Palacín, Horst von Bernuth, Renate Krueger, Isabelle Meyts, Ulrich Baumann, Maria Kanariou, Bodo Grimbacher, Fabian Hauck, Dagmar Graf, Luis Ignacio Gonzalez Granado, Seraina Prader, Ismail Reisli, Mary Slatter, Carlos Rodríguez-Gallego, Peter D. Arkwright, Claire Bethune, Elena Deripapa, Svetlana O. Sharapova, Kai Lehmberg, E. Graham Davies, Catharina Schuetz, Gerhard Kindle, Ralf Schubert

**Affiliations:** 1 Division of Allergology, Pulmonology and Cystic Fibrosis, Department for Children and Adolescents, Goethe University, Frankfurt, Germany; 2 Division for Stem Cell Transplantation, Immunology and Intensive Care Unit, Department for Children and Adolescents, Goethe University, Frankfurt, Germany; 3 Pediatric Immunology-Hematology and Rheumatology Unit, French National Reference Center for Primary Immune Deficiencies (CEREDIH), Necker Children’s University Hospital, Assistance Publique-Hôpitaux de Paris (AP-HP), Paris, France; 4 Institute for Immunodeficiency, Center for Chronic Immunodeficiency (CCI), Medical Center - University of Freiburg, Faculty of Medicine, University of Freiburg, Freiburg, Germany; 5 RESIST - Cluster of Excellence 2155 To Hanover Medical School, Satellite Center Freiburg, Freiburg, Germany; 6 Department of Rheumatology and Immunology, Hannover Medical School, Hannover, Germany; 7 Department of Immunology, The Children’s Memorial Health Institute, Av. Dzieci Polskich 20, 04-730 Warsaw, Poland; 8 Pediatrics Clinic and Institute for Molecular Medicine A. Nocivelli, Department of Clinical and Experimental Sciences, University of Brescia and ASST-Spedali Civili di Brescia, Brescia, Italy; 9 Section Pediatric Infectious Diseases, Laboratory of Medical Immunology, Radboud Institute for Molecular Life Sciences, Radboud University Medical Center, Nijmegen, the Netherlands; 10 Department of Pediatrics, Amalia’s Children Hospital, Radboud University Medical Center, Nijmegen, the Netherlands; 11 Department of Pediatric Immunology and Rheumatology, the School of Medicine, Uludag University, Bursa, Turkey; 12 Department of Pediatrics, Children’s Hospital, Faculty of Medicine, Ain Shams University, Cairo, Egypt; 13 Center of Pediatric Immunology, Western Ukrainian Specialized Children’s Medical Centre, Lviv, Ukraine; 14 Clinical Immunology and Allergy Unit, Nottingham University Hospitals, Nottingham, UK; 15 Department of Pediatrics, Cairo University Specialized Pediatric Hospital, Cairo, Egypt; 16 Department of Translational Medical Sciences, Section of Pediatrics, Federico II University, Naples, Italy; 17 Infectious Diseases and Immunodeficiencies Unit, Service of Pediatrics, Hospital Universitario Virgen de Las Nieves, Granada, Spain; 18 Department of Pediatric Oncology, Hematology and Clinical Immunology, Medical Faculty, Center of Child and Adolescent Health, Heinrich-Heine University, Duesseldorf, Germany; 19 Centre for Child and Adolescent Health, Helios Klinikum Krefeld, Krefeld, Germany; 20 Faculty of Medicine, Department of Pediatric Immunology, Ege University, Izmir, Turkey; 21 Research Unit for Pediatric Hematology and Immunology, Division of Pediatric Hemato-Oncology, Department of Pediatrics and Adolescent Medicine, Medical University Graz, Graz, Austria; 22 Pediatric Department, Infectious Diseases and Immunodeficiencies Unit, Porto Hospital Center, Porto, Portugal; 23 Pediatric Department, Faculty of Medicine, Children University Hospital in Bratislava, Comenius University in Bratislava, Bratislava, Slovakia; 24 The Royal Hospitals & Queen’s University, Belfast, UK; 25 Pediatric Infectious Diseases and Immunodeficiencies Unit, Vall D’Hebron Research Institute, Hospital Universitari Vall D’Hebron, Universitat Autònoma de Barcelona, Barcelona, Catalonia Spain; 26 Department of Pediatric Pneumology, Immunology and Intensive Care, Charité - Universitätsmedizin Berlin, Berlin, Germany; 27 Department of Immunology, Labor Berlin Charité - Vivantes GmbH, Berlin, Germany; 28 Berlin Center for Regenerative Therapies (BCRT), Charité – Universitätsmedizin Berlin, Berlin, Germany; 29 Department of Pediatrics, University Hospitals Leuven, and the Laboratory for Inborn Errors of Immunity, Department of Microbiology, Immunology and Transplantation, KU Leuven, Leuven, Belgium; 30 Department of Paediatric Pulmonology, Allergy and Neonatology, Hannover Medical School, Hannover, Germany; 31 Department of Immunology and Histocompatibility, Centre for Primary Immunodeficiencies, “Aghia Sophia” Children’s Hospital, Athens, Greece; 32 DZIF-German Center for Infection Research, Satellite Center Freiburg, Freiburg, Germany; Centre for Integrative Biological Signalling Studies, Albert-Ludwigs University, Freiburg, Germany; 33 Department of Pediatrics, Dr. Von Hauner Children’s Hospital, University Hospital, Ludwig-Maximilians-University Munich, Munich, Germany; 34 MVZ Dr. Reising-Ackermann Und Kollegen, Leipzig, Germany; 35 Primary Immunodeficiencies Unit, Pediatrics, Hospital 12 Octubre, Complutense University School of Medicine, Madrid, Spain; 36 Division of Immunology and Children’s Research Center, University Children’s Hospital Zurich, Zurich, Switzerland; 37 Department of Pediatrics, Division of Pediatric Immunology and Allergy, Meram Medical Faculty, Necmettin Erbakan University, Konya, Turkey; 38 Primary Immunodeficiency Group, Paediatric Immunology and Haematopoietic Stem Cell Transplantation, Translational and Clinical Research Institute, Great North Childrens’ Hospital, Newcastle University, Newcastle upon Tyne, UK; 39 Department of Immunology, Dr. Negrin University Hospital of Gran Canaria, University Fernando Pessoa Canarias, Las Palmas de Gran Canaria, Spain; 40 Lydia Becker Institute of Immunology and Inflammation, University of Manchester and Royal Manchester Children’s Hospital, Manchester, UK; 41 University Hospital Plymouth NHS Trust, Plymouth, UK; 42 National Medical Research Center of Pediatric Hematology, Oncology and Immunology, Moscow, Russia; 43 Research Department, Belarusian Research Center for Pediatric Oncology, Hematology and Immunology, Minsk region, Minsk, Belarus; 44 Division for Pediatric Stem Cell Transplantation and Immunology, Clinic for Pediatric Hematology and Oncology, University Medical Center Hamburg-Eppendorf, Hamburg, Germany; 45 Great Ormond Street Hospital and UCL Great Ormond Street Institute of Child Health, London, UK; 46 Department of Pediatrics, Medizinische Fakultät Carl Gustav Carus, Technische Universität Dresden, Dresden, Germany; 47 FREEZE Biobank, Center for Biobanking, Medical Center and Faculty of Medicine, University of Freiburg, Breisacher Str. 115, 79106 Freiburg, Germany

**Keywords:** Ataxia-telangiectasia, IgA deficiency, Immunoglobulins, Immunodeficiency, Lymphopenia, Mortality

## Abstract

Patients with ataxia-telangiectasia (A-T) suffer from progressive cerebellar ataxia, immunodeficiency, respiratory failure, and cancer susceptibility. From a clinical point of view, A-T patients with IgA deficiency show more symptoms and may have a poorer prognosis. In this study, we analyzed mortality and immunity data of 659 A-T patients with regard to IgA deficiency collected from the European Society for Immunodeficiencies (ESID) registry and from 66 patients with classical A-T who attended at the Frankfurt Goethe-University between 2012 and 2018. We studied peripheral B- and T-cell subsets and T-cell repertoire of the Frankfurt cohort and survival rates of all A-T patients in the ESID registry. Patients with A-T have significant alterations in their lymphocyte phenotypes. All subsets (CD3, CD4, CD8, CD19, CD4/CD45RA, and CD8/CD45RA) were significantly diminished compared to standard values. Patients with IgA deficiency (*n* = 35) had significantly lower lymphocyte counts compared to A-T patients without IgA deficiency (*n* = 31) due to a further decrease of naïve CD4 T-cells, central memory CD4 cells, and regulatory T-cells. Although both patient groups showed affected TCR-ß repertoires compared to controls, no differences could be detected between patients with and without IgA deficiency. Overall survival of patients with IgA deficiency was significantly diminished. For the first time, our data show that patients with IgA deficiency have significantly lower lymphocyte counts and subsets, which are accompanied with reduced survival, compared to A-T patients without IgA deficiency. IgA, a simple surrogate marker, is indicating the poorest prognosis for classical A-T patients. Both non-interventional clinical trials were registered at clinicaltrials.gov 2012 (Susceptibility to infections in ataxia-telangiectasia; NCT02345135) and 2017 (Susceptibility to Infections, tumor risk and liver disease in patients with ataxia-telangiectasia; NCT03357978)

**Supplementary Information:**

The online version contains supplementary material available at 10.1007/s10875-021-01090-8.

## Introduction

Ataxia-telangiectasia (A-T) is a devastating human autosomal recessive disorder characterized by cerebellar degeneration, conjunctival telangiectasia, immunodeficiency, genetic instability, and cancer predisposition [1, 2]. Recurrent infections and aspiration contribute to lung disease leading to bronchiectasis and pneumonias and often to respiratory failure [3]. In addition, A-T patients show endocrine abnormalities, such as insulin resistance, liver disease, and growth retardation [4–8]. The prevalence of patients with A-T in Europe is estimated to be 1 in 150,000. The life expectancy of patients with “classical” A-T is only between 15 and 25 years of age [9]. The major cause of death is progressive lung disease and malignancies such as lymphoma or acute leukemia [3, 9]. To date, no curative therapy is available for A-T.

It is known that deficiencies in both humoral and cellular immunity exist in A-T [10, 11]. Frequent findings include IgA and IgG-subclass deficiencies and impaired antibody response to a variety of bacterial and viral antigens [12, 13]. Lymphopenia of B- and T-cell subsets with diminished cellular immunity have been detected in in vivo and in vitro analyses [10, 11]. T-cell functional defects compromise T-cell activation and proliferation [12], abnormalities in the T-cell receptor (TCR) repertoire [14, 15], and defects in early TCR signaling events [16, 17]. These deficiencies have been described even in young A-T patients, and no deterioration of immune function has been detected in the older A-T patients [13, 18].

There is considerable clinical variation between patients with A-T, and it is becoming evident that the clinical phenotype of A-T is correlated to the presence of residual ATM kinase activity which protects the patient from the more severe “classical” disease course with early death around 20 years of age [19, 20]. Apart from residual ATM kinase activity, possible other factors, such as modifying genes and environmental factors, may contribute to a milder course of disease in some phenotypes of A-T [2].

Disease progression of A-T is demonstrable at different organ levels which are neurological decline, progressive lung disease, and liver disease [8]. Disease progression in all organs may be caused by multiple factors of which inflammation and oxidative stress play a dominant role [21–24]. The underlying mechanisms of disease progression are based on lack of major ATM functions. The major ATM functions comprise (1) ATM-dependent DNA damage response and regulation of DNA repair, (2) regulation of cell signaling and apoptosis, (3) telomere maintenance, (4) ATM-dependent response to oxidative stress, (5) mitochondrial homeostasis, and last (6) an involvement in cellular protein turnover. Thus, ATM-negative cells (neuron, lung, and liver cells) are unable to counteract inflammation and oxidative stress [22, 23, 25].

From the clinical perspective, even in classical patients without residual ATM activity, the clinical course is highly variable, A-T patients with IgA deficiency seem to show more symptoms of immunodeficiency including a higher rate of granulomas and may have a poorer prognosis [3, 26]. It was reported in earlier studies that A-T represents many immunological different conditions [9, 19]. Evidence for this includes the widely different clinical features and course of patients like the growing appreciation of the significance of raised levels of IgM (hyper IgM phenotype) [27, 28], the presence of IgG subclass deficiency [19], and in rare cases a concomitant severe combined T-cell defect with a very poor prognosis [29]. Recently, van Os et al. [30] reported that patients with the hyper IgM phenotype and patients with an IgG_2_ deficiency showed decreased survival compared to patients with normal IgG, respectively. In addition, the same group found that classical A-T patients with the ATM c.3576G > A mutation had a milder clinical phenotype in terms of prolonged survival and lower susceptibility to the development of malignancies and respiratory disease [30].

The potential immunopathogenic mechanisms with reference to this clinical heterogeneity are often not clear but have resulted in a plethora of possible mechanisms in those patients with mild or severe clinical phenotype. This prompted us to analyze the clinical history and immunological data of patients with classical A-T with and without IgA deficiency who attended the Frankfurt Goethe-University Hospital and the mortality data of the European Society for Immunodeficiency (ESID) registry.

## Patients and Methods

### Data Ascertainment

The data were collected (1) from the ESID registry and (2) from two non-interventional clinical trials at the Department for Children and Adolescence, Goethe-University, Frankfurt.

**Fig. 1:**
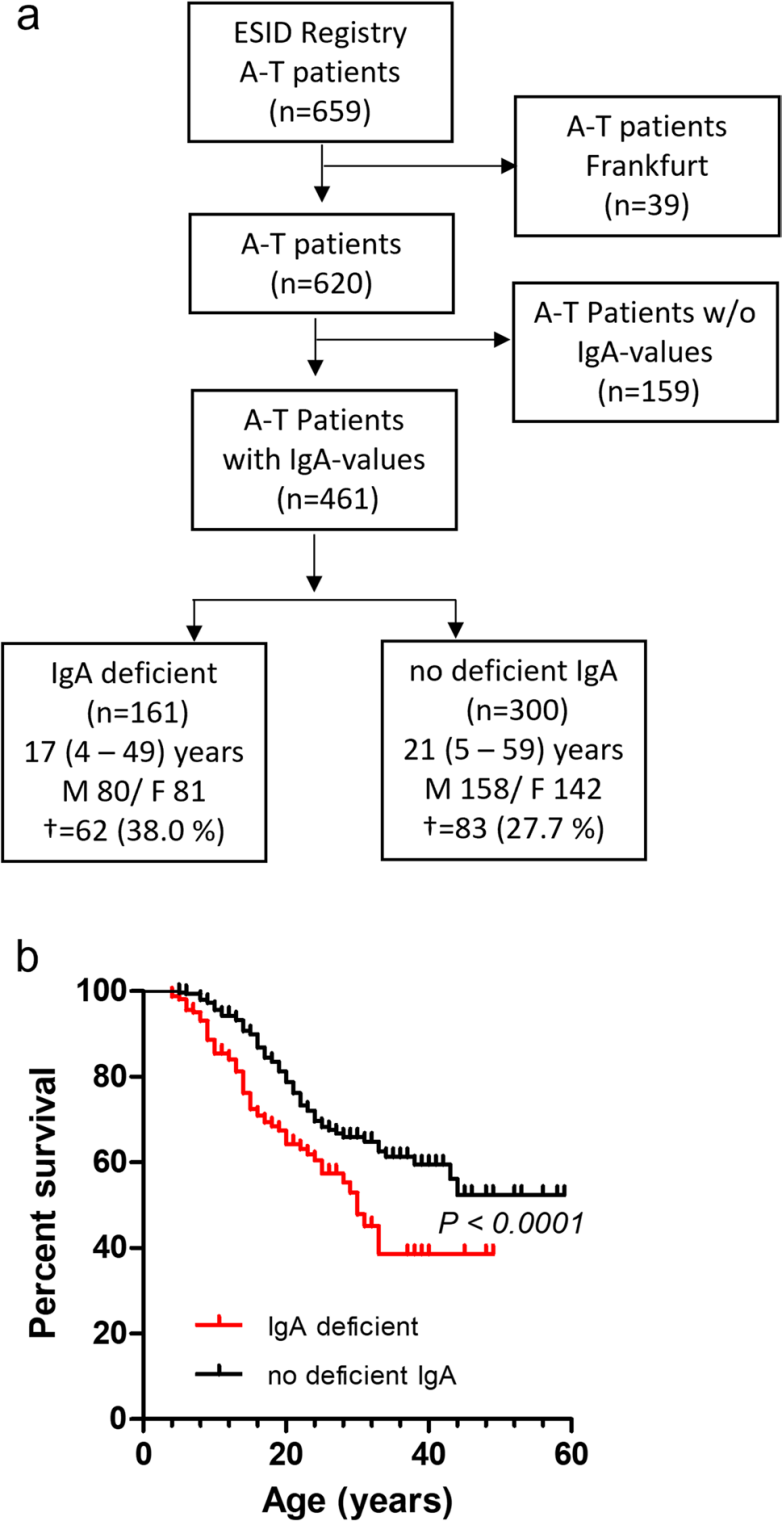
IgA deficiency influences survival in A-T. **a** Flow chart of the analyzed patients from the ESID registry. **b** Survival of patients with deficient IgA and patients with no deficient IgA. IgA < 0.07 g/L was defined as IgA deficient, and IgA ≥ 0.07 g/L was defined as no deficient IgA values below the age-appropriate normal range were defined as partial. ^†^ number of deceased patients

1. Data of 659 A-T patients on age, gender, immunoglobulin levels IgA, IgG_2_, IgM, and lymphocyte counts, date of birth, and date of death (until 2014) were collected from the ESID registry and analyzed for mortality. To avoid that patients are analyzed twice, patients from the Frankfurt cohort were excluded from the ESID cohort. A-T Patients were divided following their IgA status as deficient (IgA < 0.07 g/L) and no deficient (IgA ≥ 0.07 g/L) [3, 14]. The Ig level of the last data entry in the ESID registry was determined, and Kaplan–Meier analysis was used to calculate the survival function from their corresponding lifetime data. Survival analysis was also performed for patients with combined IgG_2_ (< 0.3 g/L) and IgA deficiency and with combined lymphopenia (< 1,000 cells/µL) and IgA deficiency and compared to patients with IgG_2_ deficiency and lymphopenia with no deficient IgA, respectively [31, 32]. An IgA (0.07 to < 0.3 g/L, ≤ 12 years and ≥ 0.3 to < 0.7 g/L, > 12 years) and IgG_2_ (> 0.3 to < 0.9 g/L) g/L value below the age-appropriate normal range was defined as partial [14].
2. Both non-interventional clinical trials were registered at clinicaltrials.gov 2012 (Susceptibility to infections in ataxia-telangiectasia; NCT02345135) and 2017 (Susceptibility to Infections, tumor risk and liver disease in patients with ataxia-telangiectasia; NCT03357978). These studies included 66 patients with classical A-T, aged two to 39 years, with a clinically and/or genetically confirmed diagnosis of A-T. Patients with IgA deficiency (*n* = 35) were compared to A-T patients without deficient IgA (*n* = 31), which composed of 24 patients with normal IgA and seven patients with partial IgA levels. The A-T patients were diagnosed based on clinical criteria and alpha-fetoprotein (AFP) values, according to recent ESID recommendations (AT Diagnostic guidelines, ESID https://esid.org/Working-Parties/Clinical-Working-Party/Resources/Diagnostic-criteria-for-PID2). Written informed consent from patients or caregivers was obtained from each subject. The study was conducted following the ethical principles of the Declaration of Helsinki, regulatory requirements, and the code of Good Clinical Practice. The study was approved by the responsible ethics committees in Frankfurt. Retrieved parameters included patient growth chart and clinical findings, as well as blood parameters such as blood count, lymphocyte subpopulation count, immunoglobulin levels in serum (IgA, IgG and IgG subclasses, IgM), and AFP. Of the 66 A-T patients from the Frankfurt cohort that are listed in the manuscript, 39 patients were recorded in the ESID registry (Fig. 1a).

### Classification into Classical and Variant A-T

The data of the ESID registry are collected by pediatricians and pediatric immunologist, and most patients (> 95%) were entered in the registry before the age of 9 years. Taken this into account, it seems very unlikely that patients with variant A-T were included in the ESID registry. Nevertheless, since ATM kinase activity was not measured in most A-T cases of the ESID registry, we performed a sensitivity analysis of survival and excluded all patients who had their 1st visit after the age of 9 years and who were older than 37.5 years to minimize the presence of A-T variants (Fig. S3).

In keeping with this approach, our pediatric center in Frankfurt, who is caring for 66 classical A-T cases in Germany, analyzed ATM kinase activity in the lab of AM Taylor, Birmingham, UK. So far, we did not detect residual ATM kinase activity in any our 66 reported cases [7, 33].

### Immunoglobulins and Lymphocytes

For a deeper insight into the immune status and to look for differences in cellular immunity between A-T patients with and without IgA deficiency, we examined blood samples from the Frankfurt patient cohort. Serum IgG levels with subclasses, IgA and IgM, AFP as well as peripheral lymphocytes were routinely analyzed at the University Hospital.

Lymphocyte phenotyping was performed in a lyse-no-wash procedure using 100 µL of whole EDTA blood per tube. The absolute numbers of CD3^+^ T-cells, CD3^+^CD4^+^ helper T-cells, CD3^+^CD8^+^ cytotoxic T-cells, CD3^−^CD56^+^ natural killer cells, and CD19^+^ B-cells were determined with tetraCHROME combinational mAb reagents for CD45-FITC/CD4-PE/CD8-ECD/CD3-PC5 (B3821F4A/SFCI12T4D11/SFCI21Thy2D3/UCHT1) and CD45-FITC/CD56-PE/CD19-ECD/CD3-PC5 (B3821F4A/N901/NKH-1/J3-119/UCHT1). Detection of naïve and memory T-cells was achieved introducing the following fluorochrome labelled antibodies: CD45RA-FITC (ALB11), CD45RO-PE (UCHL1), CD3-ECD (UCHT1), CD62L-PC5 (DREG56) CD4- (T4), or CD8-PC7 (T8). For the determination of regulatory T-cells (Tregs) as CD4^+^CD25^+^CD127^neg/dim^ cells, the antibodies CD3-FITC (UCHT1), CD127-PE (R34.34), CD4-ECD (T4), and CD25-PC7 (2A3) were used. All antibodies were conjugated with FITC, phycoerythrin (PE), phycoerythrin texas red (ECD), phycoerythrin–cyanine 5.1 (PC5), and phycoerythrin–cyanine 7 (PC7), respectively. All reagents are purchased from Beckman Coulter Immunotech (Marseilles, France) except CD25-PC7 (BD, Biosciences, Heidelberg, Germany). Measurements were performed on a Beckman Coulter FC500 five-color flow cytometer (Beckman Coulter, Krefeld, Germany). Absolute cell counts were calculated from the percentage values using a dual-platform approach. Flow-Set™ Fluorospheres served to set up the photo-multiplier tube values weekly. Stained Cyto-Comp™ Cells were applied to compensate the fluorescence overlap. The flow-cytometer optical alignment and the fluidic stability were tested daily using Flow-Check™ Fluorospheres. Immuno-Trol™ control cells were applied for verification.

### Immune Profiling by TCR-ß CDR3 Repertoire Analyses

The composition of the T-cell receptor reflects the immune competence of the peripheral T-cell compartment. To figure out constraints of A-T patients compared to healthy individuals, we performed high-throughput sequencing of the TCR-ß CDR-3 regions using the survey level for library construction, which provides representative information about rearrangement frequency. TCR-ß CDR3 repertoire analyses were performed from the Frankfurt patient cohort. Briefly, genomic DNA was extracted from peripheral blood samples using a column-based kit (QIAmp, Qiagen), and up to 2 µg DNA were utilized for library generation. Rearranged TCR-ß gene segments were amplified by multiplex PCR, and resulting products were finally extended by barcode and adaptor sequences during a 2nd PCR (ImmunoSEQ hsTCRB Kit, Adaptive Biotechnologies). High throughput sequencing (HTS) was run on a MiSeq equipment using V3 reaction Kits (Illumina) with up to 25 barcoded samples in parallel (12 pM loading concentration, 5% PhiX control). All samples were processed in duplicates, controls only once. Sequence data were analyzed with the online tool ImmunoSEQ Analyzer (Adaptive Biotechnologies) to acquire template counts (total and productive), productive clonality score on the base of Shannon entropy, and CDR3 length [34]. Calculated diversity index was obtained by dividing the number of unique rearrangements by the number of total templates [35].

### Statistics

Basic descriptive statistics and statistical analyses were performed using GraphPad Prism 5.0 (GraphPad Software, San Diego, CA, USA). Values are presented as median (range) and were analyzed using the Student’s t test, or for multiple comparisons, the one-way ANOVA with repeated measures was used. In case of not normally distributed data, the corresponding non-parametric testing was performed. The survival times of the patient groups were used to generate Kaplan–Meier survival curves which were compared using the Gehan-Breslow-Wilcoxon method, which gives more weight to deaths at early time points. Differences in survival between the groups were evaluated using the Cox proportional hazard model to calculate hazard ratios (HR) with 95% CIs. P < 0.05 was considered as statistically significant.

## Results

### Patient Data from the ESID Registry

The ESID registry comprised 659 A-T patients of which 39 patients from the Frankfurt cohort were excluded (Fig. 1a). Of these 620 patients, 461 patients had data available for IgA with a complete dataset for age, gender, and lifetime. Of these in turn, 161 patients were IgA deficient, and 300 patients had no deficient IgA with normal (*n* = 141) or partial (*n* = 149) IgA levels (Fig. 1a, Table 1). Ten patients exhibited increased IgA values. Comparison of patients with and without IgA showed that beside IgA levels, none of the other immunoglobulins were different between the groups. It is important to note that median age was significantly lower in patients with IgA deficiency (Table 1).

**Table 1:** A-T patients from the ESID-registry

	All patients	no deficient IgA (IgA ≥ 0.07 g/L)	IgA deficient (IgA < 0.07 g/L)	P value
No. of patients (n)	461	300	161	–
Age (years)	19 (4–59)	21 (5–59)	17 (4–49)	0.0001
Sex (M/F)	238/223	158/142	80/81	n.s
IgA (g/L)	0.25 (0.0–20.8)	0.70 (0.07–20.8)	0.06 (0.0–0.069)	0.0001
IgG (g/L)	9.04 (0.08 – 31.5)	9.1 (0.08 – 31.05)	9.03 (0.08–21.2)	n.s
IgG_2_ (g/L)	0.69 (0.0–33.7)	0.63 (0.0–30.0)	0.85 (0.0–33.7)	n.s
IgG_4_ (g/L)	n.d	n.d	n.d	
IgM (g/L)	1.561 (0.08–55.5)	1.57 (0.08–55.5)	1.70 (0.10–26.7)	n.s
Lymphocytes (cells/µL)	1560 (120–17,330)	1600 (120–17,330)	1495 (140–12,190)	n.s
α-feto-protein (ng/mL)	145 (1.89–1190)	158 (1.89–1190)	123 (4.71–973)	n.s
CRP	n.d	n.d	n.d	
Granulomas	n.d	n.d	n.d	

^*^ included patients with partial IgA-D (*n* = 149)

Of the 461 patients who had data available for IgA, 171 patients also had data available for IgG_2_, 458 patients for IgM, and 388 patients for total lymphocyte counts (Table S1). IgA deficiency was found in 34.9% and IgG_2_ deficiency in 29.2% of the patients. IgA levels were partial in 32.3%, IgG_2_ levels in 25.2%, IgM levels in 3.7%, and lymphocyte counts in 47.7% of the patients. Increased IgA-levels were found in 2.2%, IgG_2_ levels in 5.8%, IgM levels in 17.2%, and lymphocyte counts in 3.4% of the A-T patients.

### Frankfurt Patient’s Characteristics

From the Frankfurt cohort, 35/66 (53%) patients, median 10 years, aged from 1 to 38 years, of whom 19 males and 16 females presented with IgA deficiency, 31 patients exhibited no deficient IgA, median age 15 years ranging from 2 to 39 years, 16 males and 15 females. Of them 24 patients had normal and seven patients had partial IgA-levels (Table 2). Beside IgA, all other immunoglobulin and AFP levels were not different between the groups. Interestingly, A-T patients with IgA deficiency had a significant higher number of episodes with a significant elevation of CRP > 2 mg/dL and suffered more often from cutaneous granulomas and recurrent pneumonia (Table S3).

**Table 2:** A-T patients from the Frankfurt cohort

	All patients	no deficient IgA (IgA ≥ 0.07 g/L)	IgA deficient (IgA < 0.07 g/L)	P value
No. of patients (n)	66	31	35	–
Age (years)	12 (1–39)	15 (2–39)	10 (1–38)	n.s
Sex (M/F)	35/31	16/15	19/16	–
IgA (g/L)	0.06 (0.002–2.25)	11.0 (0.3–2.25)	0.05 (0.002–0.06)	0.0001
IgG (g/L)	8.70 (0.66–21.5)	8.34 (3.96–21.5)	9.01 (0.66–20.6)	n.s
IgG_2_ (g/L)	0.69 (0.11–6.98)	0.64 (0.12–2.76)	0.82 (0.11–6.98)	n.s
IgG_4_ (g/L)	0.02 (0.00–0.42)	0.03 (0.00–0.42)	0.02 (0.00–0.23)	n.s
IgM (g/L)	1.53 (0.17–5.45)	1.67 (0.72–5.45)	1.19 (0.17–2.73)	n.s
Lymphocytes (cells/µL)	1335 (180–3660)	1608 (595–3660)	1142 (180–2700)	0.01
α-feto-protein (ng/mL)	278 (28.7–1264)	266 (28.7–1264)	332 (49–1044)	n.s
CRP^§^	18	4	14	0.05
Granulomas	8	1	7	0.058

^*^ included patients with partial IgA-D (*n* = 7), all of them aged above 12 years of age and an IgA level > 0.3 g/L. § number of episodes with a significant elevation of CRP > 2 mg/dL

### Survival Analysis

Of the 461 A-T patients of the ESID registry with available IgA-values, 161 patients exhibited an IgA deficiency, whereas 300 showed normal (*n* = 141), partial (*n* = 149), or increased (*n* = 10) IgA levels. As shown in Fig. 1, A-T patients with deficient IgA died significantly earlier than patients with no deficient IgA (p < 0.0001). The corresponding hazard ratio (HR) for patients with IgA deficiency compared to those with IgA-levels greater than 0.07 g/L was 1.9 (1.34–2.77).

**Fig. 2:**
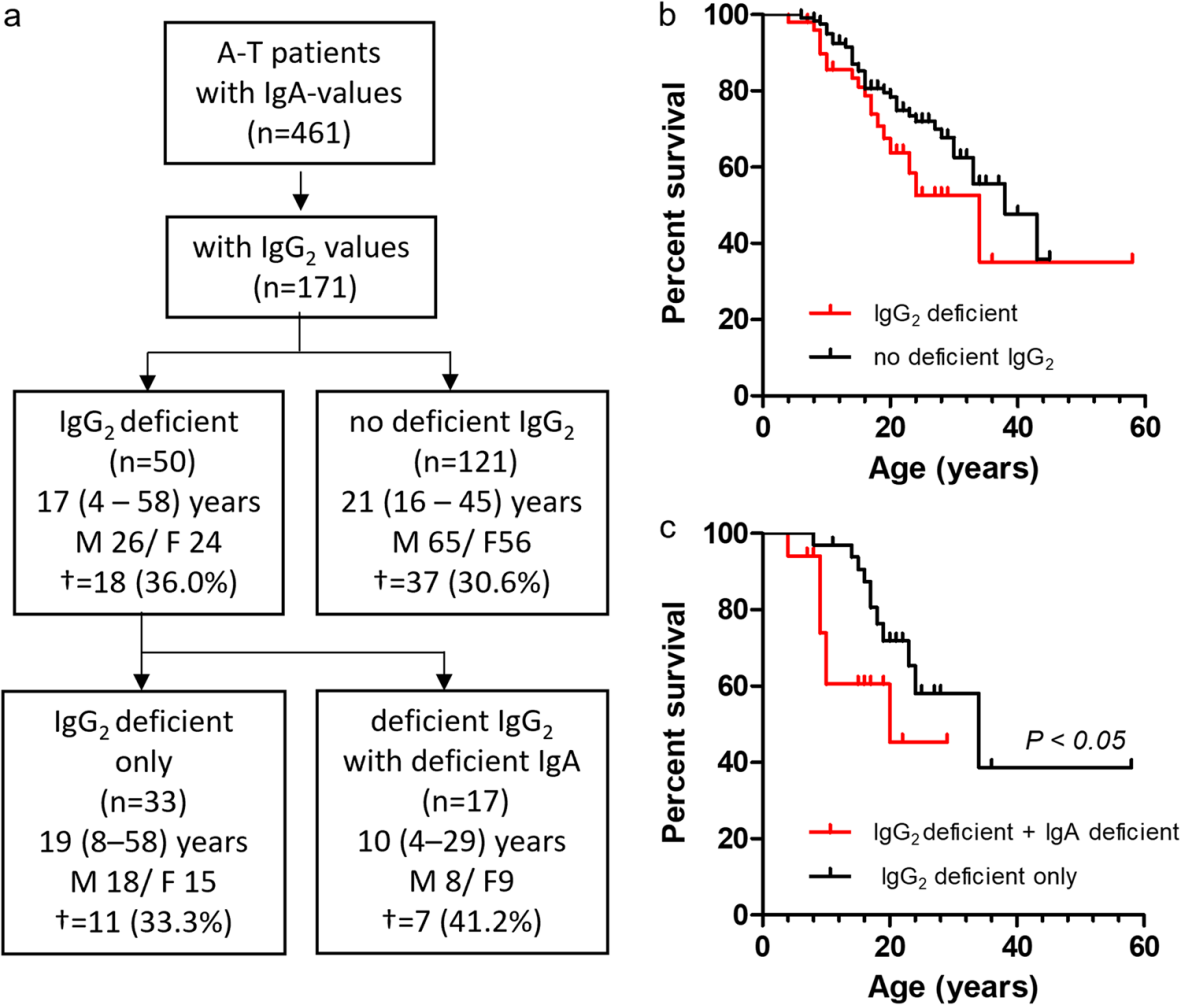
Effect of IgA deficiency on survival of A-T patients with IgG_2_ deficiency. **a** Flow charts of the analyzed patient cohorts. **b** Survival of patients with deficient IgG_2_ and patients with no deficient IgG_2_. **c** Survival of patients with deficient IgG_2_ only and patients with deficient IgG_2_ and deficient IgA. IgA < 0.07 g/L, and IgG_2_ < 0.3 were defined as deficient. ^†^ number of deceased patients

Fifty out of 171 A-T patients exhibited a deficient IgG_2_ (Fig. 2). This group of patients showed no difference in mortality rate compared to patients with no deficient IgG_2_ levels with a HR of 0.9 (0.3–1.5). In contrast, A-T patients with both IgG_2_ deficiency and IgA deficiency showed a higher mortality than patients with IgG_2_ deficiency and no deficient IgA values (HR 2.9, 0.9–9.2).

**Fig. 3:**
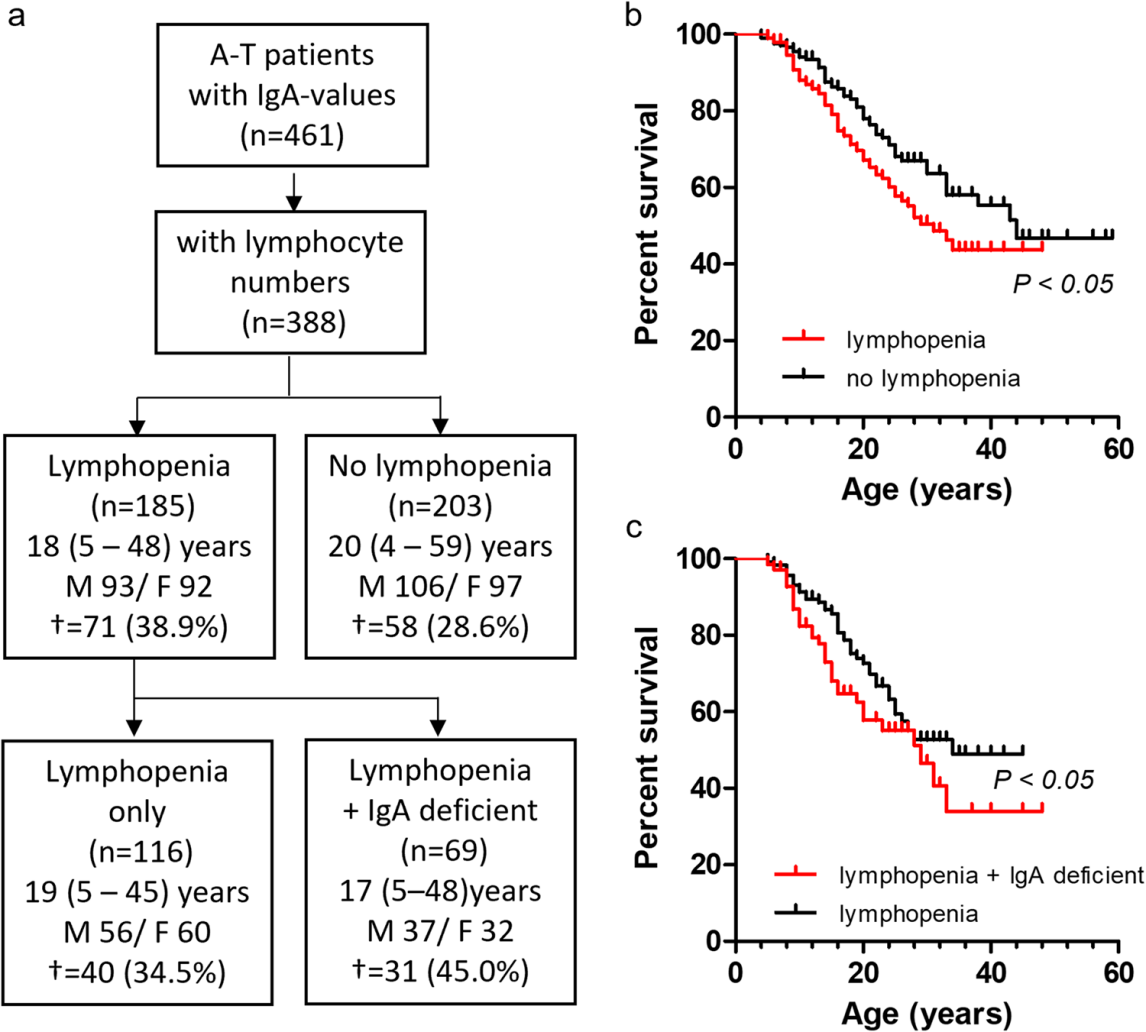
Effect of IgA deficiency on survival of A-T patients with lymphopenia. **a** Flow charts of the analyzed patient cohorts. **b** Survival of patients with lymphopenia and patients with normal lymphocytes. **c** Survival of patients with lymphopenia only and patients with lymphopenia and deficient IgA. IgA < 0.07 g/L was defined as deficient. Lymphopenia was defined as ≤ 1,500 cells/µL. ^†^ number of deceased patients

Analysis of the ESID registry data revealed further that 185 patients out of 388 suffer from lymphopenia (Fig. 3). Patients from this group died at a younger age than patients with normal lymphocyte counts with a HR of 1.5 (1.1–2.2). Death rate was found further increased when patients exhibit lymphopenia together with an IgA deficiency (HR 1.5 (0.9–2.5)).

### Ig-Replacement Therapy

In the ESID registry data about therapeutic treatments were available from 298 patients. Treatments included Ig-replacement (204), antibiotics (61), and others (antidepressant (1), anti-reflux drug (1), cytostatic (1), growth hormones (1), H2 receptor blocker (1), steroid (2), vitamins (3), iron (1), neurological (1), immunostimulant (1), and immunosuppressant (1)) (Fig. S1). No differences could be detected in the percentage of Ig-replacement therapy between patients with IgA deficiency and patients with no deficient IgA.

### Cause of Death

Data about the cause of death was available from 60 A-T patients with available IgA values in the ESID registry and the patients from the Frankfurt cohort (Fig. S2). Of these, 27 died from respiratory failure, 29 died from cancer, and 4 died for other reasons. While little more patients with no deficient IgA died from respiratory failure (respiratory failure 52.9%; cancer 44.1%), the group with IgA deficiency exhibited a slightly higher proportion of patients who died from cancer (respiratory failure 43.5%; cancer 56.5%).

### Decreased Lymphocyte Subpopulations in A-T Patients with IgA Deficiency

**Fig. 4:**
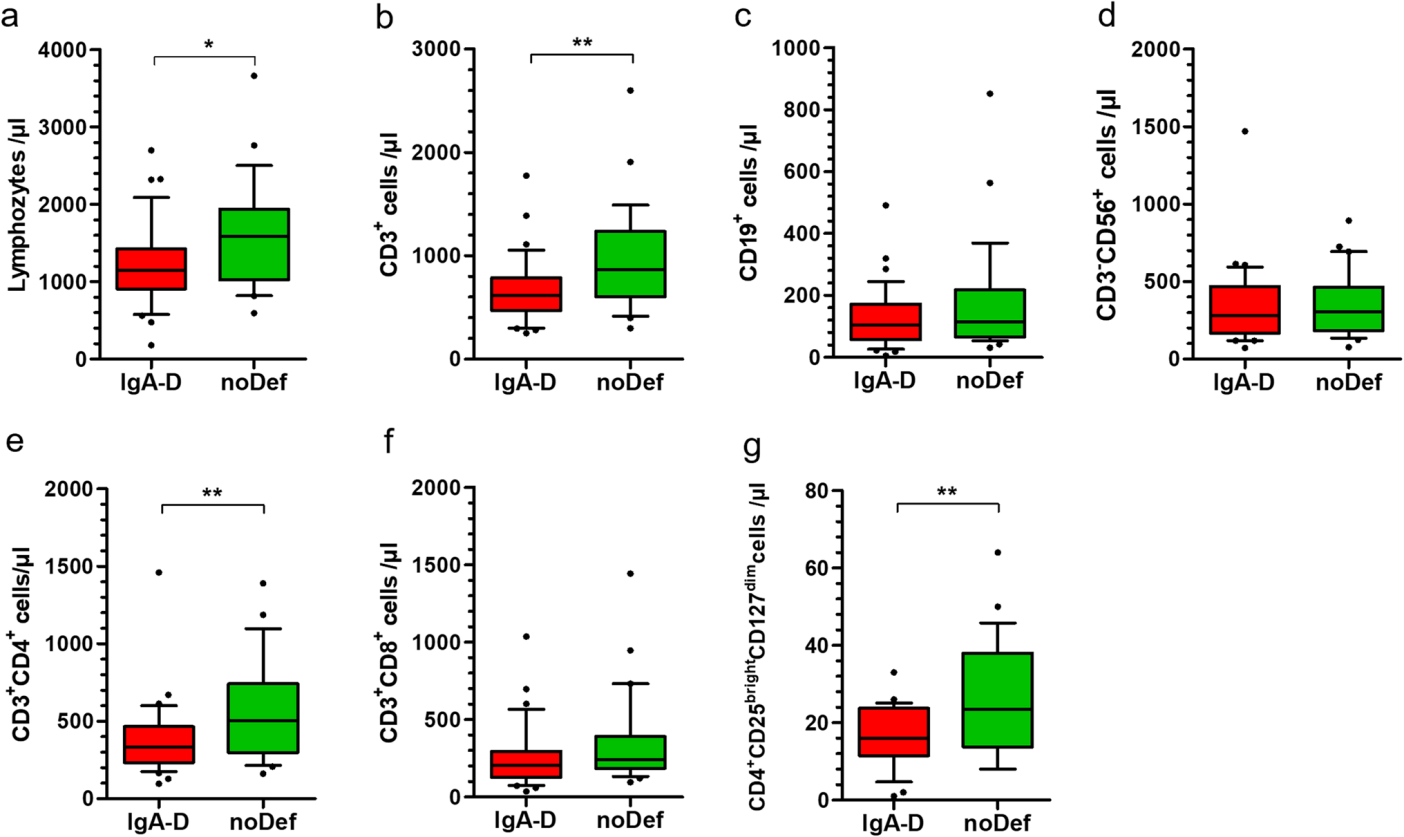
Lymphocytes in A-T patients with deficient IgA (IgA-D) and no deficient IgA (noDef). Blood samples of A-T patients were analyzed for total numbers of **a** CD3^+^ T-cells, **b** CD19^+^ B-cells, **c** CD3-CD56^+^ NK-cells, **d** CD3^+^CD4^+^ helper T-cells, **e** CD3^+^CD8^+^ cytotoxic T-cells and **f** CD4^+^CD25^bright^CD127^dim^ regulatory T-cells. IgA-D. * p < 0.05, ** p < 0.01

Patients with A-T have significant alterations in their lymphocyte phenotypes [11] . Subsets of CD3, CD4, CD8, CD4/CD45RA, and CD8/CD45RA cells were significantly diminished compared to age matched standard values. In A-T patients with IgA deficiency, total numbers of lymphocytes were significantly decreased (1148 cells/µL, 180–2700) compared to patients with no deficient IgA (1592 cells/µL, 595–3660, p < 0.01). This was evident particularly for CD3 T-cells (IgA-D: 613 cells/µL, 251–1775; noDef: 865 cells/µL, 299–2599; p < 0.01), for helper T-cells (IgA-D: 335 cells/µL, 97–1459; noDef: 505 cells/µL, 162–1389; p < 0.01), and for regulatory T-cells (IgA-D: 16 cells/µL, 1–33 noDef: 24 cells/µL, 8–64; p < 0.05) but not for cytotoxic T-cells, B-cells, and NK-cells (Fig. 4).

**Fig. 5:**
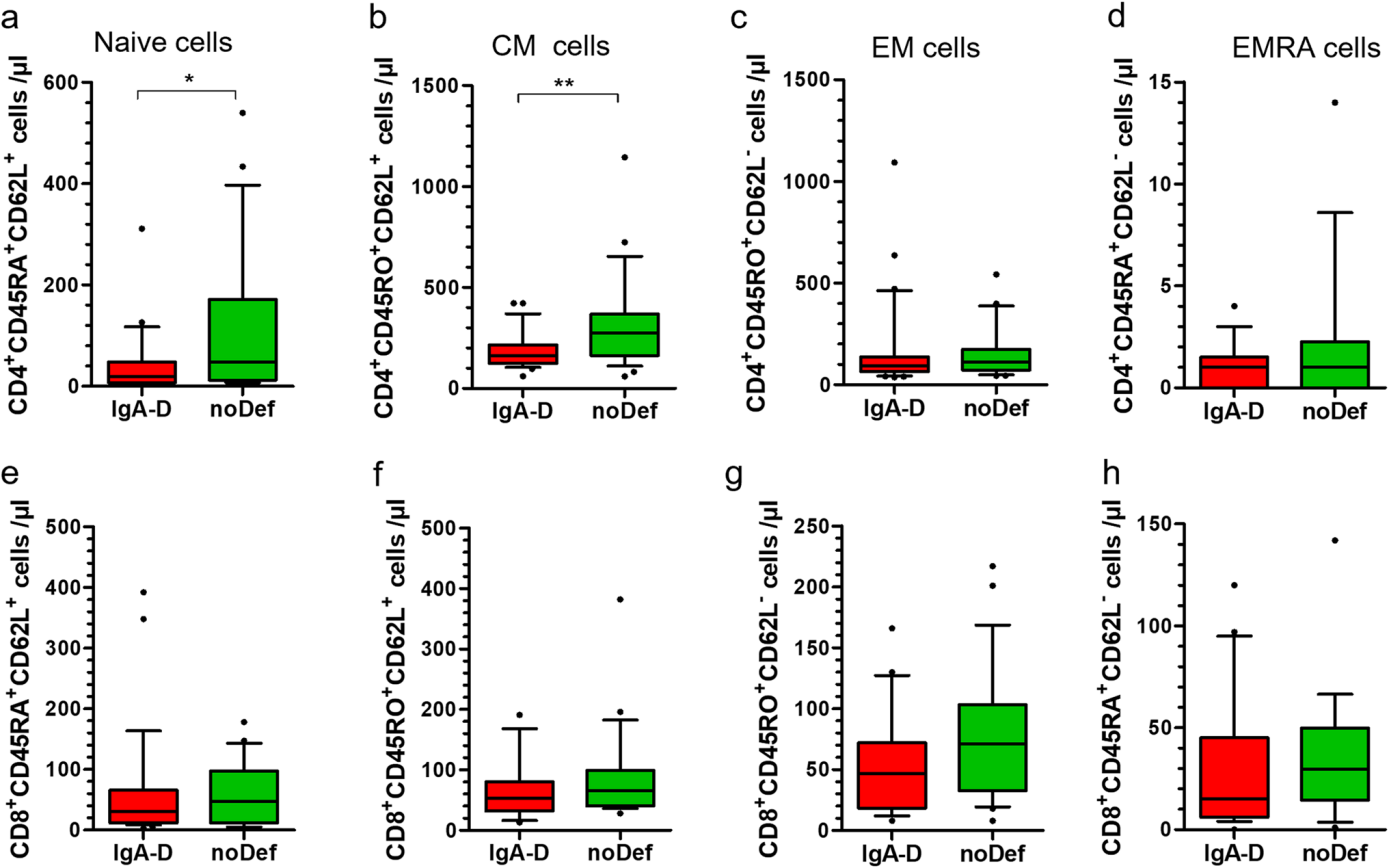
T-cell subpopulations in A-T patients with deficient IgA (IgA-D) and no deficient IgA (noDef). Blood samples of A-T patients were analyzed for total numbers of **a** CD4^+^CD45RA^+^CD62L^+^ naïve T-cells, **b** CD4^+^CD45RO^+^CD62L^+^ central memory (CM) T-cells **c** CD4^+^CD45RO^+^CD62L^−^ effector memory (EM) T-cells, **d** CD4^+^CD45RA^+^CD62L^−^ effector memory RA (EMRA) T-cells, **e** CD8^+^CD45RA^+^CD62L^+^ naïve T-cells, **f** CD8^+^CD45RO^+^CD62L^+^ central memory (CM) T-cells **g** CD8^+^CD45RO^+^CD62L^−^ effector memory (EM) T-cells, **h** CD8^+^CD45RA^+^CD62L^−^ effector memory RA (EMRA) T-cells. * p < 0.05, ** p < 0.01

Differences in CD4 T-cells were found in the naïve CD4^+^CD45RA^+^CD62L^+^ (IgA-D, 19 cells/µL, 1–311; noDef, 48 cells/µL, 6–540; p < 0.05) as well as in the central memory CD4^+^45RO^+^62L^+^ (IgA-D, 164 cells/µL, 60–422; noDef, 274 cells/µL, 59–1145; p < 0.01) T-cell subpopulation (Fig. 5).

### T-cell Receptor Rearrangements

**Fig. 6:**
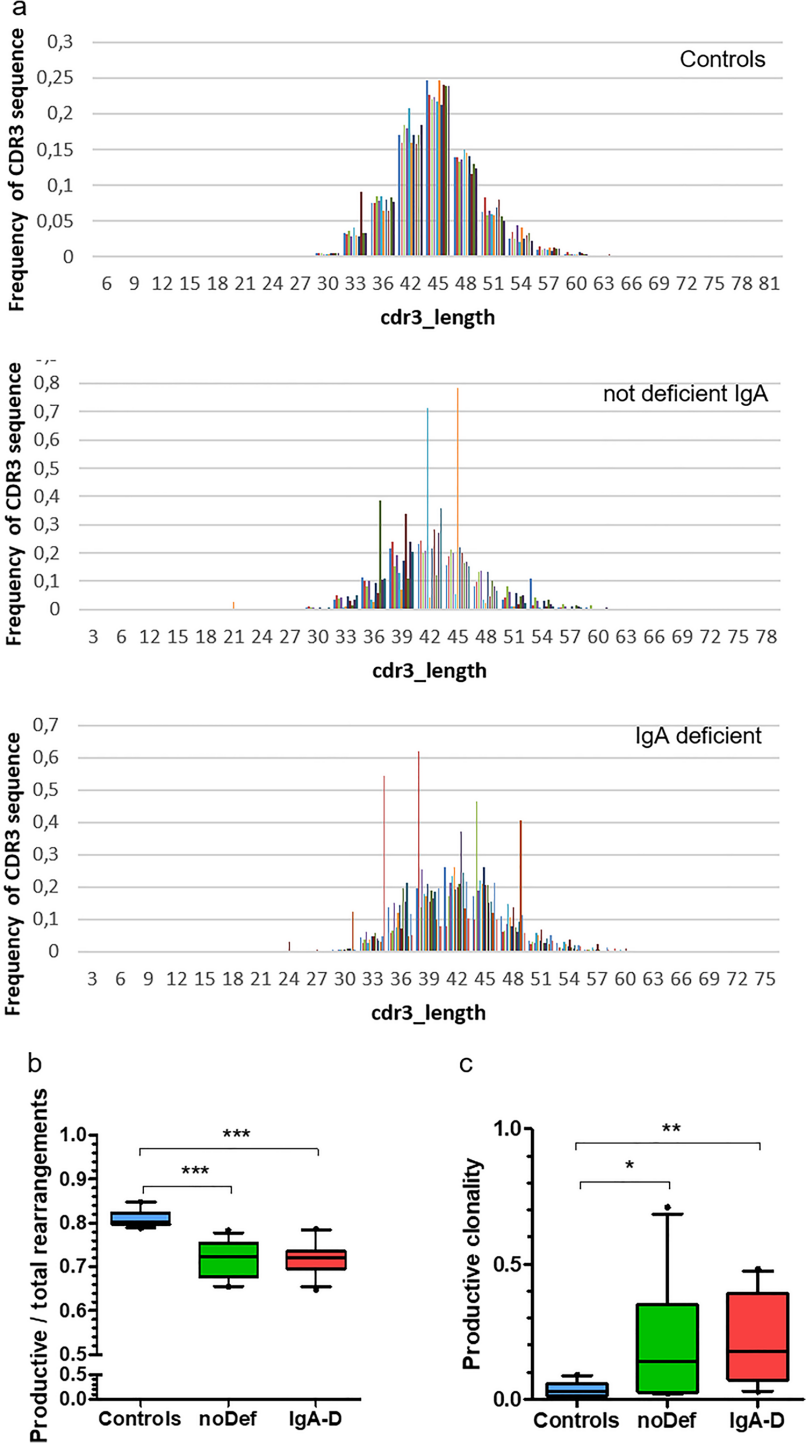
T-cell rearrangement in A-T patients with deficient IgA (IgA-D), no deficient IgA (noDef) and controls. **a** CDR3 length distribution of the TCR-ß repertoire given as relative frequency (total = 1) for control, noDef, and IgA-D samples. **b** Calculated diversity index of controls and both patient groups. **c** Productive clonality score of controls and both patient groups. The clonality score is derived from the Shannon entropy, which is calculated from the frequencies of all productive sequences divided by the logarithm of the total number of unique productive sequences. This normalized entropy value is then inverted (1 — normalized entropy) to produce the clonality metric. Entropy was calculated by summing the frequency of each clone times the log (base 2) of the same frequency over all productive reads in a sample

We evaluated the mean template numbers and range of 23,470 (5514 to 55,205), 18,626 (4195 to 41,586), and 21,507 (9332 to 32,357) for case, cohort, and control, respectively. Beneath that, we identified unique productive rearrangement numbers and range of 7211 (1253 to 13,687), 7249 (1394 to 25,601), and 12,802 (6374 to 18,151) in the same order as before. Both patient groups show affected TCR-ß repertoires characterized by the diminished portion of functional rearrangements and by tendency of clonal expansion (Fig. 6a–c).

## Discussion

At present, no curative therapy for A-T exists, but new treatment options like stem cell therapy, infusions of erythrocyte-delivered dexamethasone and antisense oligonucleotide (ASO) are emerging [2, 33, 36]. Thus, markers of disease progression and long-term outcome are urgently needed to define patients with the poorest prognosis who might be considered first for such new treatments. The major interest of the present study is to investigate the clinical and immunological consequences of IgA deficiency in A-T and whether IgA is a possible marker to assess the prognosis of A-T patients. Therefore, we analyzed the mortality data of 659 A-T patients of the European Society for Immunodeficiency (ESID) registry as well as the clinical history and immunological data of patients with classical A-T with and without IgA deficiency of our Frankfurt patient cohort. Our study confirmed earlier findings that immunodeficiency is closely related to decreased survival in A-T [19, 27]. This is particularly evident for A-T patients with IgG_2_ and IgA deficiency. In contrast to the study of van Os et al. which found similar survival for A-T patients with and without IgA deficiency, our data provide strong evidence for an influence of IgA deficiency on survival in A-T [19]. These contradictory results might be explained by the much larger cohort of 461 A-T patients analyzed in the present study. In contrast to Os et al., no differences in survival between A-T patients with and without IgG_2_ deficiency were seen. This finding could be based on the proportion of patients with concurrent IgA deficiency. Indeed, IgA deficiency significantly reduced survival of A-T patients with IgG_2_ deficiency and further reduced survival of patients with lymphopenia. The lack of difference in survival of patients with IgA and IgG_2_ deficiency in the Frankfurt cohort might be explained by the relatively low numbers. In addition, none of the patients in the Frankfurt cohort died on respiratory failure before the age of 20 years.

Abnormalities of immunoglobulin profiles and defective polysaccharide antibody synthesis are well known in A-T [11, 12, 19, 37, 38]. In our large cohort, IgA and IgG_2_ deficiencies were found in 34.9 and 29.2% of patients, whereas the hyper IgM phenotype was present in 17.2% of patients only. The pathogenesis of the hyper IgM phenotype, IgA and IgG_2_ deficiency is still unknown; however, a defective terminal differentiation of B-cells may be responsible. Recently, it was shown that inactivation of the DNA damage response pathway like in A-T cells leads to an impaired class switch recombination [39, 40].

While our data show that presence of IgA is associated with a better survival, the basic mechanisms involved are not clear. In general, IgA deficiency is often found in healthy blood donors with a defect in the differentiation of IgA cell-producing plasma B-cells but without any harm of such individuals [41]. The functions of IgA at the body surface are well known. IgA forms a barrier against pathogens by neutralizing bacterial products and impedes pathogens to penetrate the mucosal epithelium [42]. In addition, IgA is downregulating inflammatory cell responses via FcαRI and may act as an important non-inflammatory regulator of mucosal immunity [43]. The important role of IgA is supported by the fact that several bacteria have developed escape mechanisms by producing IgA1 proteases or molecules that hamper interaction with IgA receptors [42]. Our finding of an elevated number of episodes with increased CRP in patients with IgA-deficiency supports the protective role of IgA in inflammatory processes.

The underlying mechanisms leading to IgA deficiency are multifarious and anomalies in lymphocytic apoptosis, cytokine networking, and costimulatory signaling, and the presence of predisposing MHC complex alleles has been described [44]. In A-T patients, it has been shown by Driessen et al. that antibody deficiency is the result of a disturbed B- and T-cell homeostasis and that naïve CD4^+^ T-cell numbers are closely correlated with CD27^+^IgA^+^ memory B-cells [14]. Phenotyping of our cohort of classical A-T patients revealed that patients with IgA deficiency had significantly lower lymphocyte counts compared to A-T patients without IgA deficiency due to a further decrease of naïve CD4 T-cells (CD4/CD45RA), whereas no differences in absolute B-cell numbers could be detected. T-cell imbalance was accompanied with lower central memory CD4 cells (CD4^+^45RO^+^62L^+^ cells) and regulatory T-cells. This is an interesting finding in regard of T-cell help to B-cells. Central memory (CM) T-cells home secondary lymphoid organs such as B-cell-enriched follicles and germinal centers to provide help to B-cells [45]. Some of these CM T-cells express CXCR5 and have been defined as T-follicular helper (Thf) cells. They induce B-cell differentiation and class-switching by ligation of CD40Ligand as well as T-cell-derived cytokines [46]. Another population of T-cells that augment the germinal center response are T follicular regulatory (Tfr) cells [47]. Tfr cells are a unique subset of Treg cells that are localized in the germinal center (GC) of the B-cell follicle. Thus, beside disturbed B-cell homeostasis, an incomplete T-cell help program could be responsible for reduced IgA levels in A-T patients with IgA deficiency. In addition, we compared the mutations of our patients with and without IgA deficiency. As shown in the supplement no distinctive patterns were identified. This is in line with previous reports and confirms that no correlation exists between ATM mutations and immunoglobulin phenotype [19, 48, 49].

In light of old and new emerging treatments, patients with classical A-T need to be better characterized on the immunoglobulin and molecular level. Immunoglobulin replacement and antibiotic prophylaxis could be helpful for patients with recurrent infections and bronchiectasis [50]. There is a phase 3 trial ongoing (Ataxia-Telangiectasia Trial with the EryDex SysTem) to evaluate the efficacy, safety, and tolerability of EryDex to prevent neurological decline in A-T (Erydel https://www.erydel.com/News/April 14, 2020). Results of this large trial may be available in autumn 2021. Hematopoietic stem cell transplantation (HSCT) is an encouraging approach to correct immunity and prevent the development of hematologic malignancies [33]. However, to what extent the restored immune system and the increase of ATM protein may prevent neurological decline and the development of other malignancies is not known. In addition, the first patient is currently treated by an antisense oligonucleotide to block progression of neurodegeneration by injections in the spinal fluid. At present, the long-term outcome needs further evaluation.

Our study has several limitations; these include the fact that it was a retrospective analysis and not a prospective or randomized clinical trial. In addition, we analyzed two cohorts, the ESID data and the Frankfurt cohorts, which differ in several biological aspects. The distribution of IgA deficiency (ESID cohort (IgA-D 161/noDef 300) and Frankfurt (IgA-D 35/noDef 31) was statistically different (p = 0.009). The reasons for these differences are hardly to explain but may be due to a well-known bias of the ESID registry. Mainly pediatric immunologists who care for patients suffering from frequent infections and a more pronounced immunodeficiency collect the data of the ESID registry. Thus, more healthy patients are not entered in the registry. Nevertheless, to overcome these differences of the cohorts, we analyzed survival only in the ESID cohort and removed all Frankfurt data from this analysis. Vice versa, the ESID registry does neither provide data about lymphocyte subsets such as T- and B-cell numbers nor data about T-cell rearrangement, these parameters were analyzed solely in the Frankfurt cohort. Although both cohorts differ in their ratio of IgA deficient and no deficient patients, the groups are well comparable in respect of the genetic background and the distribution of the other immunoglobulins and therefore well suited for the analyses performed. Moreover, as shown in the results section, the dataset of 659 registered A-T patients were rather incomplete. Only 461 patients had data available for IgA and only 171 patients for IgG subclasses. Maybe since IgG subclass analysis is a more sophisticated and expensive method than IgA measurement. The large imbalance of data might affect our Kaplan Meier curves of survival. Also, we must admit that the cause of the underlying mortality was rarely reported, and no corresponding mutations were known for patients in the registry.

IgA deficiency is difficult to diagnose in children < 4 years according to international consensus [51]. However, the age range 5–61 years of the registered patients ruled out that the absence of IgA was simply related to the young age of patients. To further increase the specificity of our finding, we chose the criteria deficient IgA and partial IgA in comparison to normal IgA values. Finally, we cannot completely rule out that some “A-T variants” with residual ATM kinase activity may affect our finding sinceATM kinase activity was not measured in most A-T cases of the ESID registry. To overcome this drawback, we did a sensitivity analysis and excluded all patients who were not registered before the age of 9 years and who were older than 37.5 years to further minimize the presence of A-T variants, since A-T variants may develop symptoms much later than classical A-T cases [52]. The sensitivity analysis confirmed that A-T patients with absent IgA exhibit a lower survival rate than A-T patients without IgA deficiency.

In conclusion, for the first time our data show that patients with IgA deficiency have significantly lower lymphocyte counts and subsets, which is accompanied with reduced survival compared to patients without IgA deficiency. IgA, a simple surrogate marker, is indicating the poorest prognosis for classical A-T patients.

## Supplementary Information

Below is the link to the electronic supplementary material.

Supplementary file1 (DOCX 242 kb)

## Data Availability

The datasets for this manuscript are not publicly available due to the respective agreements with the documenting centers. Requests to access anonymized datasets should be directed to the ESID registry, e.g., via the corresponding author.
