# Supplementary material for: Simple Measurement of IgA Predicts Immunity and Mortality in Ataxia-Telangiectasia
Source: J Clin Immunol. 2021 Sep 3;41(8):1878–92. doi: 10.1007/s10875-021-01090-8 (PMC8604875; doi:10.1007/s10875-021-01090-8)
Supplement: Supplementary file 1 — Supplementary file1 (DOCX 242 kb) [file 10875_2021_1090_MOESM1_ESM.docx]

Supplementary Material

# Supplementary Tables

**Table S1 Number of A-T patients from the ESID-Registry with their respective immunoglobulin phenotype**

|  | **deficient** | **partial** | **normal** | **increased** | **total** |
| --- | --- | --- | --- | --- | --- |
| **IgA*** | 161 | 149 | 141 | 10 | 461 |
| **IgG** | --- | 125 | 305 | 30 | 460 |
| **IgG_2_*** | 50 | 43 | 68 | 10 | 171 |
| **IgM *** | --- | 17 | 362 | 79 | 458 |
| **Lymphocytes**** | --- | 185 | 191 | 12 | 388 |

*Ig values below or above the age-appropriate normal ranges were defined as deficient, partial and increased, respectively. IgA <0.07 g/L, IgG_2_<0.3 g/L were defined as deficient. IgA ≥0.07 - <0.3 g/L, ≤12 years and ≥0.3 - <0.7 g/L, >12 years and IgG_2_ >0.03 - <0.9 g/L were defined as partial.

**Lymphocytes <1500 cells/µL were defined as partial.

**Table S2 Characteristics of A-T patients from the Frankfurt cohort**

**Table S3 Clinical characteristics of IgA deficient and no deficient IgA patients**

|  | All patients | | no deficient IgA | | IgA deficient |
| --- | --- | --- | --- | --- | --- |
| Number of patients | | 66 | | 31 | 35 |
|  | |  | |  |  |
| Age (years) | | 14 + 10 | | 15.6 +10.4 | 13 + 9 |
| Sex (M/F) | | 35/31 | | 16/15 | 19/16 |
| Granuloma | | 8 | | 1 | 7^§^ |
| Recurrent Pneumonia | | 27 | | 9 | 18^§^ |
| Bronchiectasis* | | 9 | | 4 | 5 |
| IgG replacement | | 25 | | 8 | 17 |
| Death due to lung failure | | 8 | | 6 | 3 |
| Cancer | | 15 | | 7 | 8 |
| Death due to Cancer | | 8 | | 4 | 6 |
| Liver disease | | 18 | | 6 | 12 |
| Diabetes | | 4 | | 3 | 1 |

Values are presented as Mean + SD; Liver disease was defined as GPT >50U/L; Diabetes was defined as HbA1c >5.7%; *Bronchiectasis proven by CT scan

§ Patients with IgA-D suffers significantly more from granuloma and recurrent pneumonia

## 2 Supplementary Figures


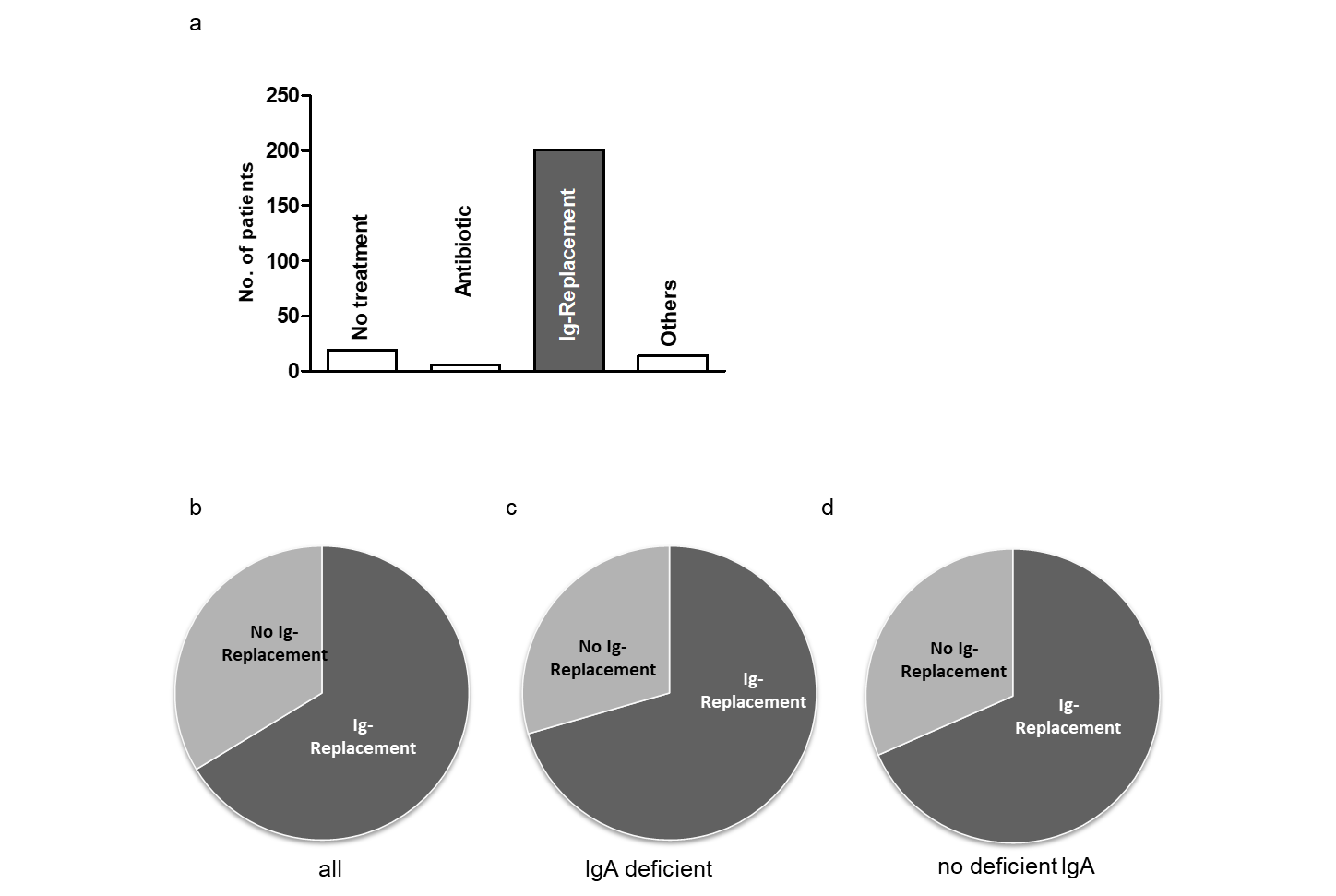


**Fig. S1** Ig-replacement therapy. Therapeutic treatments of A-T patients from the ESID registry. **a** Columns show the different treatments included Ig-replacement, antibiotics, and others (antidepressants, anti-reflux drugs, cytostatics, growth hormones, H2 receptor blockers, steroids, vitamins, iron, immunostimulants, and immunosuppressants). Circle diagram presents the percentage distribution of Ig-replacement therapy and no-replacement therapy of **b** all patients with treatment data, **c** patients with deficient IgA, and **d** patients with no deficient IgA.


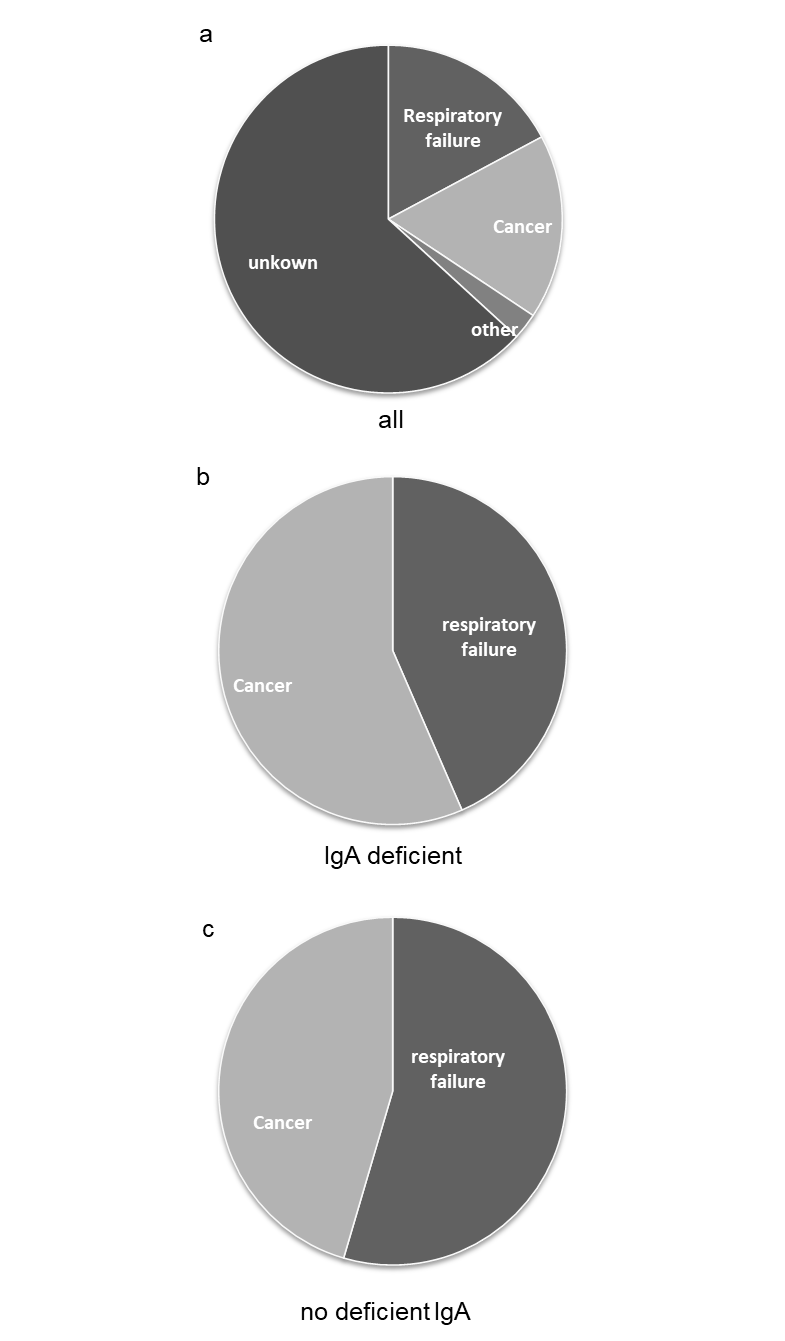


**Fig. S2** Cause of death. Cause of death of A-T patients from the ESID registry. Circle diagram presents the percentage distribution of respiratory failure, cancer, and others of **a** all patients, **b** patients with deficient IgA, and **c** patients with no deficient IgA.


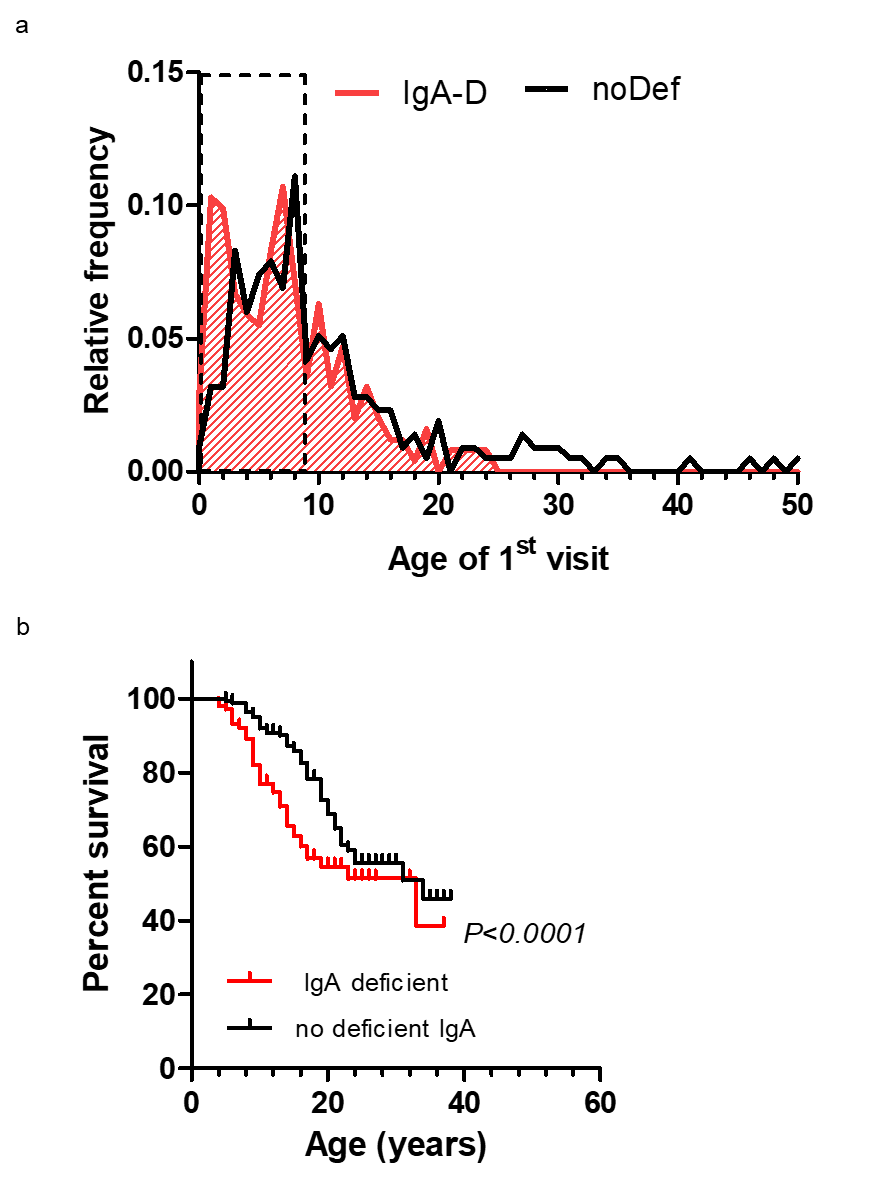


**Fig. S3** Sensitivity analysis to minimize influence of variants on survival of A-T patients with and without IgA deficiency. **a** Relative frequency of patient’s age at first visit in the ESID Registry (IgA-D, IgA deficiency (red line); noDef, no deficient IgA (black line). The hatched square presents the patients with the first visit before the age 10. Only this group was included in the survival analysis, since A-T variants may develop symptoms much later than classical A-T cases [52]. **b** Survival of A-T patients who were registered before the age of 10 years and who were not older than 37.5 years with deficient IgA or with no deficient IgA. IgA <0.07 g/L was defined as deficient.
